# Surgery

**Published:** 1857-09

**Authors:** 


### Bi-Monthly Periscope.

### SURGERY.

1. Treatment of Aneurism by Manipulation.—Since the report of Mr. Fergusson's case (Med. Times and Gazette, Nov. 15th, 1856), of aneurism of the subclavian artery, in which he proposed a new method of treatment, which consisted in squeezing the tumor, with a view of breaking up a portion of its fibrinous contents, and forcing it to the distal end of the tumor, or the artery leading from it, we notice two cases cured by the same plan, which we subjoin. The first is reported by Mr. Robert Little (Med. Times and Gazette, May 23d), and the second by Prof. George C. Blackman, of Cincinnati (Western Lancet, June, 1857).

"D. M., an albino, aged 53, admitted into the County Donegal Infirmary on the 6th October, 1855, with an aneurism of the right subclavian artery, gives the following history of his case: States that having been in the habit of dealing in eggs and fish, which he usually carried through the country in a basket suspended on his back by means of straw ropes, through which he passed his arms, he first felt pain in the right arm in the preceding month of March, which gradually became so severe, that in the month of May he was frequently obliged to sit down on the roadside and remove his burden for a time. Soon afterwards he discovered a tumor above the right clavicle, directly corresponding to the site on which one of the ropes pressed, which also became painful after a short time; and in the beginning of July he perceived 'a beating in the lump,' which then began to enlarge rapidly. In the month of August he says, he had such a feeling of drowsiness, that for a fortnight he slept the greater part of each day and night, during which time he lost his appetite and took nothing but milk, and at this time he was unable to bend his fingers. Sleep then suddenly deserted him, and he declares that for a fortnight prior to his admission into the Infirmary he did not sleep a single hour, owing to the intensity of the pain in the tumor and along the arm.

"Symptoms on Admission.—A tumor equal in size to the largest goose egg occupies nearly the entire of the supra-clavicular region, extending from the clavicular attachment of the sterno-cleido-mastoid to the acromial end of the clavicle, which has a strong pulsatory movement, that is visible from the most remote part of the ward, and is accompanied with a loud *bruit de soufflet;* it is soft and compressible, and is red and somewhat inflamed on the surface; from which circumstances Dr. Greer, under whose notice the patient first came, greatly feared the aneurism would have burst. There is no appreciable dulness on percussion under right clavicle, but the respiratory murmur is not as distinct as on the opposite side however, this may arise from its being somewhat masked by the loud bruit on that side; the superficial veins of head and neck are considerably enlarged, but he does not suffer either from cough, dyspnœa, or dysphagia; tongue tolerably clean, pulse at wrist 80, and regular; appetite not good. His chief source of complaint is a severe and constant pain extending from the tumor down the right arm as far as the tips of the fingers, which he says is most acute about the middle of the humerus, and he is constantly compressing this part with the other hand, conceiving that it gives him some relief. At first he got sedatives, had cold applied to the aneurism, and each night had a full anodyne, which treatment somewhat moderated the violence of the pulsation, and made him feel more comfortable, and after a few nights, when the anodyne had been considerably increased, he got some tranquil rest.

"In December, he was bled twice from the arm, and ice was kept constantly applied over the tumor for three weeks, without any manifest improvement, except that the redness and inflammatory appearance of the integument covering the aneurism have completely disappeared; in other respects, the symptoms remain unaltered. Having seen the report of Mr. Fergusson's very interesting case, I resolved to follow his suggestion in this apparently hopeless one, and I must confess I did so without any very sanguine expectation of success. Accordingly, on the 1st of January, 1856, by making gentle but steady pressure with my thumbs alternately over the aneurismal sac, I succeeded in displacing some of the coagula, and directing them towards the distal end of the artery. No other local treatment was adopted, but he was ordered the persesquinitrate of iron internally. For the first two days no change was perceptible in either the tumor or the arm; but at the third day the pulse at the wrist was manifestly weaker, and the arm somewhat colder than the opposite one. These symptoms gradually increased up to the tenth day after the manipulation of the sac, when no pulsation could be felt in either radial, brachial, or axillary arteries. The tumor itself had now become more solid, and the bruit and pulsation were both diminished; the violent pain in the tumor and along the arm has also decreased, but now he complains of a sensation of coldness over the right shoulder and scapula, and of a severe pain extending along the side of the neck and back of the head, which increased in severity for a month, and the arm became greatly wasted, and partially paralyzed, retaining very little sensation and scarcely any power of motion.

"*March.* All pulsation in the aneurism having now ceased to be visible, pressure was applied over it.

"*November.* Both bruit and pulsation have completely disappeared; the aneurism is not more than one-third its original size, and is quite solid; the anterior edge of clavicle feels thin and sharp, from the absorption of its upper surface, caused by the pressure of the sac, and the pain alongside of head and neck, heretofore so much complained of, has completely subsided. The arm has regained its natural temperature, and, although still considerably attenuated, he can use it tolerably well, sensation having also returned to it. A very slight pulsatory wave can now be felt in the radial artery, but not in either brachial or axillary. Two superficial arterial branches, of considerable magnitude, can also be traced, running in a transverse direction across the remains of the aneurism, one immediately above the clavicle, the other somewhat higher up.

"*March*, 1857. Having again admitted the patient into the Infirmary within the last few days, for the purpose of examining his condition, the absorption of the tumor is steadily progressing, being now not larger than a walnut. Pulse at the wrist somewhat stronger than at last report, but still not to be felt in either brachial or axillary. Sensation and motion are completely restored to the arm. He is free from all pain, and says he feels perfectly well, and intends resuming his former occupation. I may mention that most of my medical brethren in this locality having taken a deep interest in this case, and visited him from time to time while under treatment, have also seen him since his last visit to the Infirmary, and agree with me in considering the cure to be most satisfactory and complete."

"John Austin, æt. 28, a native of England, entered the Commercial Hospital on the 7th of April. Four months previously, he felt a sharp pain along the course of the femoral artery at the junction of the lower and middle third of the thigh, and for the first time he observed a pulsation in this region. He had worked for many years as a file-cutter, and had been accustomed to use a small anvil, which was held between his thighs. A swelling was soon detected, and this continued to increase until the time of his admission. There was a space of about three inches between the upper margin of the tumor and Poupart's ligament, and measured along the axis of the limb, the swelling was five inches at its base. The aneurismal bruit was very distinct, and the pulsations perceptible across the amphitheatre. Compression at the groin caused the tumor to diminish considerably in size, and it would immediately regain its former dimensions when the pressure was removed. The patient complained of numbness and other painful sensations in the knee, leg, and foot. As the tumor was daily increasing, and as there was no other indication of disease of the arterial system, I determined to bring the patient under the influence of veratrum viride, in order to subdue the force of the circulation. From the time of his admission he was kept on a low diet, and cathartics were administered. On the 11th, I ordered six drops of the tincture every three hours. On the morning of the 12th, I found that the pulse had been reduced in frequency from 94 to 65. At 10 o'clock A.M. of this day, he was brought before the class, when with my thumb I pressed forcibly on the aneurismal sac, for the purpose of dislodging a portion of the fibrinous layers, hoping thus partially to obstruct the artery, and to favor the further deposition of fibrine in the sac. Skey's tourniquet was now applied with moderate force between the tumor and Poupart's ligament. The progress of the case may be learned from the following record kept by Dr. N. J. Sawyier, the House Surgeon:

"At 12 M. his pulse being 110, full, strong, and bounding, he was bled ℥ix. Pulse came down to 50, soft and regular, and continued low for several days. (The following are extracts from the Case-Book):

"*April* 13, A.M. Suffers no pain nor uneasiness at all; slept well last night. Entire limb diminishing rapidly in size. Kept the apparatus tight. General health good; whenever any untoward symptom arose, it was promptly met, and the patient kept in a good condition. At intervals, the shooting pain was felt in the tumor, but it gradually subsided altogether.

"17*th*. Prof. Blackman ordered the tourniquet to be taken off, the bandage reapplied from the toes, up over the tumor, upon which it was to be tightly wrapped, and the patient to be bled, after which the following was administered: ℞—Antimon. and potass. tart. gr. ¼; pulv. opii. gr. ⅛. Sig.—Take every three hours. Patient's pulse came down to 65, soft and regular.

"19*th*. *Souffle* ceased entirely, but the pulsation continues, though it is very weak.

"22*d*. Pulsation in tumor has ceased altogether.

"25*th*. Is in fine spirits; has no pain, and wants to walk about. General health very good.

"30*th*. Has walked some steps, and complains of nothing but weakness.

"*May* 21. The pulsation in the tumor has never returned. The femoral is firmly plugged as far as the origin of the profunda, and in the popliteal space the pulsation of the artery is hardly perceptible. The tumor is daily decreasing in size, and the patient is anxious to leave the hospital and resume his business."—*American Journal of the Medical Sciences*, July, 1857.

2. PROFESSOR BOECK ON SYPHILIZATION.—It is a fact well known to all, that M. Auzias Turenne, in trying to induce syphilis in animals, observed that they, after a number of inoculations, became, as it were, proof against the syphilitic virus. He called the state which was induced in the body by the name of syphilization, and M. Sperino, being made acquainted with the observations of M. Auzias, immediately adopted the idea of using a continued inoculation with the virus in order to cure syphilis, an idea which was so speedily realized, that it seems as if both had at the same time used syphilization in men. At first the thing looked so absurd, that all concerning syphilization was considered as mere fancy. Most did not find it worth thinking of, and thought right to reject it only from theoretical reasons, as if experimenting for one's self, or even looking at the experiments of others, would make one an accomplice in spreading fantastic dreams; such being the opinion generally held as to the doctrine of M. Auzias Turenne. Now the matter has changed. Medical men have already ventured, in more places than one, to repeat the experiments of M. Auzias; and it has been found that what was termed nonsense was only the plain truth. A new proof has been given, that it is better to be cautious in judging of anything *à priori*, as nature does not always follow the way that we point out to her in our study. On the contrary, she wills that we shall find out her ways, however difficult it may be for us.

As is often the case, one only saw the dark side of the question. The chief points of it were forgotten in the absurd idea of preserving everybody from an illness which is, in general, only for those who attract it to themselves. It was forgotten that the system, after a number of inoculations with the syphilitic virus, becomes proof to it. In fact, persons had been to that degree irritated against the said abuse of syphilization, that they did not think of the physiological side of the inquiry, which was so much the more extraordinary, as it was not here the question of any theory but of a fact, of which everybody might convince himself. It can be readily shown that, after a number of inoculations, it is at length impossible to produce any effect. The same result then ensues, whether the inoculation be made with the syphilitic virus or with water. The organism, after such a number of inoculations, seems to have acquired the same relation to syphilis as it does to vaccine lymph after a single inoculation with this latter poison. This fact has been proved in more than a hundred persons who have been treated here by syphilization, so that I must consider it unnecessary to enter more closely upon that matter. This immunity takes place gradually, the ulcers being smaller after every succeeding inoculation, until at length the pustules are quite abortive, or the result is an absolutely negative one. This diminishing by degrees may suffer some modifications, on which I shall, however, not dwell here. I have spoken of them before. The result—complete immunity—always will take place; it will take place in all individuals. This, is a rule without exception. The immunity may take place earlier or later, the susceptibility for the syphilitic virus being different in different individuals ; and besides, I suppose the intensity of the syphilitic poison to be dfferent—a circumstance that will occasion a considerable difference in the time necessary to produce the immunity.

Every physician of any experience must have observed that mercury is far from being a satisfactory remedy. I will not speak about the mercurial treatment being disagreeable to the patient, especially when those preparations are used which produce salivation—it is so inconvenient to the sick person, that he is obliged to be within doors, to be in bed; that after the treatment he is very sensible to cold and humid air. All this would not be worth mentioning, if mercury was a certain remedy, and harmless to the constitution ; but this is not the case. Mercury is so very far from being a certain cure, that, on the contrary, it is a doubtful question whether mercury is at all a remedy for syphilis. This doubt is no new one. It is not brought forward just now in order to support another line of treatment. Authorities on the disease have long held it. I shall only name Ricord and Cullerier. But let us not protect ourselves with authorities; let us hold to that which every day's experience proves. For instance, let us see what is the proportion of relapses in our hospital at Christiania. By closely examining the reports, we have found that the number of relapses is about twenty-five per cent. of the whole number of individuals treated. If now we remember that a great number of relapses do not take place until after some years—that many of these relapses never come to be treated in this hospital—that very often we find syphilis developed to its highest degree in children, whose parents have been treated with mercury, and are apparently in good health—that we often find relapses, in the form of osseous affections, in the surgical wards; of apoplexies, and incomplete and complete paralysis, in the medical wards of the hospital; and in the form of mental maladies in our asylum for the insane—then I should think the percentage number will be a very large one. I might very well add here the many scrofulous and tuberculous affections which, in my opinion, certainly originate in syphilis—nevertheless, I do not mean to say that I think syphilis to be exclusively the cause of these maladies; but by doing so I should enter on debatable ground, which is not at all necessary for proving that mercury never produces the result we should expect from a remedy, that very often it is quite the opposite of a remedy, that it is a pernicious, a dreaded agent.

If it be evident, as I think it is, that the remedies hitherto used against syphilis are uncertain, and even pernicious, then it is not only allowable, it is our duty, to try the new one that is offered to us. To me the only question was—in what cases syphilization might be used. I have already mentioned that I always thought *prophylactic* syphilization to be an absurdity. Therefore, I shall not dwell any longer on it. The question is—whether syphilization ought to be used in all cases where syphilis exists ? This question is easily answered. I cannot predicate with certainty if all those who get primary syphilis will get constitutional disease. The simple chancre is not in general accompanied by any constitutional affection. The

Hunterian one is certainly a consequence of a constitutional syphilis, but we may easily deceive ourselves in respect to the induration. Therefore, I never use syphilization where there is merely primary syphilis. It is not until the constitutional symptoms have appeared that I consider this method allowable, for then I am convinced that I do not introduce anything into the organism but what is there before. I cannot double a malady already present. So I am quite certain not to do any harm to the patient.

This may be the fit place for mentioning shortly how I produce syphilization. Without any other preparation than a bath, or in my private practice even without this, I apply on each thigh, and on each arm, or on the sides only, three inoculations in every one of those places, with matter taken from a primary ulcer, or from an artificially produced one in a person who has been syphilized. I choose the first-named places for those who are lying in the hospital, but I inoculate the sides of those who, during syphilization, are going out attending to their business. However, I must add, that I never confine my inoculations exclusively to the sides. If they do not prove effectual there, I apply them on the thighs, on which we shall almost always find the ulcers to be larger, deeper, and of a longer duration. Therefore, I think this place the best, and never fail inoculating there. Every third day I inoculate anew. As long as the last inoculations produce pustules, I take the matter from these. In some cases I have always tried to take the virus from the first-made inoculations, thinking to find there the strongest matter, and thereby, perhaps, be able to achieve the cure in less time; but the cases in which the treatment has been accomplished in this manner are so few, that I should not venture to draw deductions from them. In syphilized children, I have only applied one inoculation on each thigh, and generally also on each side, every third day, or perhaps at longer intervals. The ulcerations produced in this manner may occasionally become phagedenic in grown-up persons. Many wounds may be united into one, and form a large ulcerating surface. This, however, does not signify in the least, provided the treatment be continued without being alarmed. The inoculations are a certain remedy against the phagedenic ulceration. In children, the ulcers are generally so small as not to cause any inconvenience. It is only in cases which have been mercurialized before that I have sometimes seen the artificial ulcerations enlarge, yet never to an alarming degree.

In some instances the inoculated person becomes proof to one sort of virus. I then take the matter for inoculation from another, preferring a case which has had a different origin; this then proves effectual. But sometimes they become proof to this also, and I then seek for a third source; and thus I go on as long as any matter at all will operate.

Moreover, it is worth noticing that immunity does not occur, and the syphilitic phenomena do not vanish, earlier in children than in grown-up persons. The time necessary to produce immunity is about three months. However, it depends upon the number of inoculations that may be employed—upon the symptoms that have taken place; and in children it seems to depend upon their syphilis having been acquired or inherited. The quality of the virus even may not be without influence. When immunity is attained, the syphilitic phenomena generally vanish. However, should this not be the case, it should cause no uneasiness, as they will certainly vanish within a short time, without any remedy being used.

It is not uncommonly the case, that during syphilization a new eruption takes place; but this always exhibits symptoms of the same nature as were observed at the beginning of the process of syphilization. These eruptions need not cause any anxiety. The operator may quietly go on inoculating, and things will proceed as in other cases. One phenomena that I have often seen develop itself under syphilization is iritis. This has been very intense in some cases; but I do not make it the subject of any special treatment, either antiphlogistic or derivative, and the result has hitherto been always favorable.

The syphilitic poison does not run a rapid course, as was known a long time before we heard anything of syphilization. We often see the constitutional symptoms not to show themselves until after some months. Therefore, there is nothing astonishing in the fact, that the curative results of inoculation do not show themselves until after some time.

But if even by syphilization alone we cannot effect a cure in all cases, it is, nevertheless, an indispensable remedy. Patients who have been nearly destroyed by syphilis and mercury may be restored by it to health. The cases belonging to this class may present very different aspects, and the effect of syphilization on them, of course, also different. I therefore think the best way to give my view of the matter is to arrange them in separate groups, viz.:—

1st. The early constitutional cases, recently treated with mercury, in which the same symptoms have reappeared. Here syphilization will, in some cases, produce as certain an effect as in cases not treated before, but we oftener find some irregularity. The phenomena vanish and return again. That which I have said takes place in the individuals not mercurialized is repeated here; namely, it is always the same forms which existed at the beginning of the syphilization that return.

2d. The affection may be still confined to the cutaneous system and the pituitous membranes, but the tubercular forms may be predominant, ulcerations on mucous membranes may go deeper, or the affection may be in the subcutaneous areolar tissue. We may even have the *tubercular serpigenous syphilide.** These affections are more slowly acted upon. The reason for this may partly be found in the fact, that these forms are often rather of old standing. Mercurial treatment, iodine, &c., have been used against them, and we also often see bad forms show themselves within a year after the primary affection. This seems to depend on individual constitution, for it often has no relation to the quantity of mercury, or the care taken of the patient during the treatment.

If, in these cases, new eruptions come out during syphilization, we shall always find them to be more superficial than the earlier affection, if even they have the same form as that which existed at the beginning of the syphilization treatment. It happens in these cases, especially, that the inoculations, after a small number of them have been made, do not produce any effect; then we must give iodine, after which we shall again have larger pustules and ulcers.

3d. Affections of the osseous system. Here syphilization hardly ever seems to produce any effect. But when iodine has been used earlier, producing results of only a short duration, then syphilization, united with iodine, seems to relieve the nocturnal pains more certainly; but osseous tumors remain unaltered by syphilization.

* Radesyge.

4th. Affections of the nervous system—hyperæsthesia and incomplete and complete paralysis—may occur:—First, In combination with other syphilitic symptoms, and in those cases I have seen them diminish under the influence of syphilization. Secondly, They may be the only phenomena left as the result of the mercurials used against the primary syphilis; and, under these circumstances, we see little or no effect from syphilization. However, I must observe, that all the cases of that sort which I have hitherto treated have been of old standing, and have for a long time been treated with iodine, &c.

5th. Mental maladies, finally, may be the result of the mercurial treatment. I have had no opportunity of employing syphilization in such cases, but I consider it well worth trying. The idea that syphilization should be the last refuge seems to be quite as if quinia should not be given in the beginning of an intermittent, but that the system should be first injured by different other medicines, and then quinia given afterwards.

As the result of the great many observations made with syphilization, it seems sufficiently proved that the syphilitic virus heals constitutional syphilis, and that it cures the malady without doing any harm whatever to the organism. On the contrary, we see that the uneasiness, the rheumatic pains which often accompany constitutional syphilis, vanish under continued inoculations.

The immediate effect of syphilization upon the organism is generally also very favorable, but there are some who have thought that it may, perhaps, operate perniciously in future time. To this I have only to say, that I can show many individuals discharged from hospital more than three years ago who have remained in uninterrupted good health, and that in not one of the persons treated in this manner, can I point out any unfortunate result whatever which could be ascribed to syphilization.

If, finally, I were to comprehend, in a few words, my opinion about syphilization used as a curative remedy, I should say—

1. Syphilization is undoubtedly useful against syphilis; it is the only certain remedy that we know, and it is not pernicious to the organism: mercury, therefore, ought to be banished as a curative remedy.

2. Syphilization is not so certainly useful against *mercurialized syphilis*, but it ought always to be tried. It often does cure it entirely, and it at least does not fail to do some good in the greatest number of cases.

3. The application of syphilization against other maladies than syphilis ought to be tried with the greatest possible care and exact observation.—*Dublin Quarterly Journal, Feb.* 1857.

3. CASE OF EXCISION OF THE OS CALCIS, BY AN IMPROVED METHOD OF OPERATING—RAPID RECOVERY. By *Clifford Morrogh*, M.D., of New Brunswick, N. J.—On the first day of October, 1854, I was requested to attend Mr. Elsworth Dansbury's son, for necrosis of the right os calcis. The patient was twelve years old, of fair complexion, and bright intellect; he was very much emaciated from the disease; both parents were healthy, and seemed robust. They gave the following history of his case:—About nine months prior to my seeing him, the boy got his foot slightly squeezed between two railroad cars; he did not complain much at first but, after the lapse of a week or two, the ankle and foot became swollen and painful. Medical assistance was sought, but the disease increased, and several abscesses broke, leaving cloacæ, which continued to discharge. The ankle continued very painful, and caused intense suffering when placed on the ground; hectic fever supervened, and the boy became peevish, fretful, and very much reduced. This was his condition when I first saw him. The integument round the ankle was livid, glazed, and tumid; it was perforated by three or four fistulous openings, which discharged an ichorous pus; various parts of the calcaneum could be reached by a probe through these openings. It was felt to be denuded and roughened; no other bone could be felt in this condition, although the swelling extended for some distance beyond its limits. I proposed the operation of excision, as a means of avoiding the humiliating alternative of amputation. The parents readily assented; and on the 24th of the same month, the operation was performed in the following manner, Dr. A. T. Taylor, of this city, Dr. Pool, of South River, and several of the patient's friends being present:—The boy was placed on his left side, on a table, and rendered insensible with chloroform. A vertical incision was made over the posterior extremity of the os calcis, extending from the superior to the inferior surface, and continued along the inferior surface of the bone, to its articulation with the cuboid, taking care to keep outside of the plantar artery. The incision was then carried upward to a short distance above the superior surface of the bone, without wounding the peroneal tendons; this incision described a square flap on the outside of the foot, which was dissected up, and the tendo-achillis separated close to its attachment. A strong narrow scalpel was then introduced under the peroneal tendons, and made to separate the calcaneo-cuboid articulation, without severing the tendons, or the artery beneath. The knife was then introduced between the upper surface of the bone and the astragalus, and made to cut the interosseous ligament, and gradually separate the articular surfaces. The calcaneum was then rotated, so as to bring its upper surface outward till the internal made its appearance, when the soft structures were carefully separated, principally by the handle of the scalpel, thus completing the operation. After this mode of proceeding, we had the satisfaction of seeing the posterior tibial and plantar arteries pulsating in perfect integrity, while the tendons of the peroneal muscles, as well as those of the flexor longus policis, flexor communis digitorum, and tibialis posticus were uninjured. No ligatures were required, the flap was approximated, a piece of flat sponge applied over the wound, and a gutta percha splint, previously moulded over the instep so as to keep the foot extended. The operation was followed by a total cessation of pain and febrile action.

The boy improved rapidly in health; the wound healed mostly by adhesion, the rest by granulation. The cavity partly filled up by what I supposed to be fibro-cellular tissue; and in two months after the operation the boy could run swiftly. At the present time, he wears a small pad in the shoe under his heel, and does not experience the least inconvenience from the deficiency of the bone.—*N. Y. Journal of Medicine* for July, 1857.

4. SELECTIONS FROM THE UNPUBLISHED MANUSCRIPTS OF THE LATE ABRAHAM COLLES, PROFESSOR OF SURGERY TO THE ROYAL COLLEGE OF SURGEONS OF IRELAND. Edited by his Son, *William Colles.*—ON DISEASES OF THE GENITAL ORGANS AND RECTUM; and on BRACHIAL ANEURISM.—In the present communication, I have selected some cases presenting peculiarities in appearances or symptoms, and sometimes leading to improper surgical interference. These I think useful to record, as they show that the surgeon cannot be too cautious before coming to a conclusion as to the nature of a disease, or the remedies to be adopted, and that he should be aware not only of the ordinary course of nature in the various affections, but should also recollect that she sometimes deviates from the regular course, and presents anomalous appearances which may puzzle the most experienced. I also add some selections and observations on cases of disease of the rectum; the first two of which present some useful suggestions.

*Paraphimosis relieved.*—CASE I.—January 24, 1814. A young lad, aged 15, had drawn back the prepuce on Thursday, and it remained so till Monday evening. I made two or three ineffectual attempts at reduction by force; then tried by squeezing the base of the glans to reduce its size; but I here also failed. I now thought I should have to divide it, and proceeded to the operation. On passing the director under the stricture, it struck me I might raise the skin, and depress the base of the glans. Passing the point of the instrument under the prepuce, at the right side, I raised it up, so as to be able to catch the skin between the finger and thumb; then, passing the director to the left side, I found that in its progress it raised up the skin, and brought it forward over the glans, and the reduction was effected without force.

CASE II.—January 10, 1815. A man with paraphimosis applied; it had occurred three or four days ago, and prevented his having any sleep for the last three nights. Immediately after connection he endeavored to draw forward the prepuce, but could not succeed. The stricture is at some distance behind the corona glandis, and is very tight; the line of stricture is ulcerated, and of a blackish color; the skin, both before and behind the stricture, is much swollen. This appeared a very bad case, but was reduced with great facility.

I made the patient lie on the floor, on his back, and, kneeling at his right side, I passed the silver director under the stricture towards the right side, and, while the point lay under the stricture, I pressed down the swollen part of the prepuce and corona glandis with the stalk of the instrument; then, pressing this portion backward, I found it was easily pushed back beyond the stricture. In this manner, passing the director from the right to the left side, I quickly succeeded in removing the paraphimosis.

I think an instrument might with advantage be employed in such cases, somewhat of the shape of a shoehorn, only not much hollowed on the surface which is to be applied to the glans penis.

I next record a case of rupture of the urethra, and another supposed rupture of the corpora cavernosa penis, in consequence of awkward, ill-directed efforts at connection.

CASE III.—*Ruptured Urethra in Coitu, fatal.*—June 10, 1812. Called to Mr. K——, aged about 38; flabby constitution, full habit, and prone to obesity. Married two months. Last night, on having connection, he hurt himself; it would seem that the penis had pushed against the bones of the pubes. At the instant he felt severe pain, and as if something had been broken; next day the integuments of the penis and scrotum were full, livid, and a small swelling was visible on the left side of the penis, anterior to the scrotum. This was hard and circumscribed. Passed urine, not more than an ounce, mixed with blood, and attended with scalding pain at the tumor, urine drawn off by catheter.

11th. The great pain attending micturition prevented his passing more than a few drops of urine; again drawn off; tumor same size.

12th. Tumor much increased in size; some fetid pus, on pressure, passed from the urethra; gum-elastic catheter passed, and retained; he was feverish; in the evening he had a severe rigor.

13th. Fever, and discharge, mixed with air, increased.

15th. Made an incision into the tumor, and let out a mixture of pus, urine, and blood mixed with air; catheter again passed and retained; rigors occur daily, increasing; pain in head; abdomen tympanitic; desponding; countenance yellow.

20th. Died unexpectedly. The urine passed by urethra and also by the opening in the abscess for the last two or three days on withdrawing the catheter.

CASE IV.—*Tumor in the Penis.*—December 12, 1808. Mr. G——, aged 38 last March, while in the act of copulation pushed against the bone of the pubes, and felt he had hurt himself; he observed a lump come on the dorsum penis, and the organ curved upwards in erection.

On examination, a flat, square lump, about the middle of the dorsum penis, was found lying under the vena superficialis penis, firm in structure; it appeared to be movable. This was, however, doubtful. This lump has increased, and, in proportion to its growth, renders the curvature in erection greater, when he feels as if the penis were bound by a cord; does not know if the tumor increases on erection.

Ordered blistering, and to take pills of hemlock and calomel.

I find also three or four cases recorded of tumors in the corpora cavernosa penis, but no cause assigned for their appearance, and no treatment successful in removing them.

The Case No. V. is worthy of being recorded. The two following cases represent the testes not in the natural position.

CASE V.—*Œdema* (*Elephantiasis*).—April, 1833.—Mr. G——, aged 48, for four years past has had a swelling of the prepuce and skin of the penis; it was not discolored till lately. The prepuce is distended, so as to form a curve anterior to the glans; the size of the penis is considerably increased, the swelling rather hard, but allows of pitting; the scrotum is also thickened anteriorly, not posteriorly; the skin of the penis is of a purple color, and shines; no pain or inconvenience, except that he cannot have connection; the general health is very good; never was in a warm climate; is a married man, with children. I only recollect one other case at all like this, in an hospital patient.

CASE VI.—*Testis misplaced.*—Yesterday I saw Mr. K——, aged 36, who had the left testis in the perineum, with one edge above, the other below; the right testis is lodged in the scrotum, and is in every respect natural; the left is at present inflamed, either from a hurt received on getting down off a public coach, or from an injury by a girl of the town; the integuments covering it are not loose, and he seems to suffer severe pain from it, particularly on the anterior spine of the ilium.

CASE VII.—Mr. S——. That portion of the integuments corresponding to the scrotum in its color and corrugation is very small, not larger than a child's, and this does not receive the testes; these are lodged in a soft, smooth, white skin at the side of the scrotum, and are of a full size; the scrotum appears as a small cone on the top of the smooth skin. He has not any children.

Those who have one testis in the abdomen generate, of which I recollect many instances; but I do not recollect an instance of those who had both testes in the abdomen generating.

I find two or three cases where a tumor on the cord was mistaken for hernia, and a truss worn for some time, but they present nothing extraordinary. The next is a case of hydrocele cured by injection of its own fluid.

Case VIII.—*Hydrocele injected with its own fluid; cured.*—September 13, 1834. This day I injected a hydrocele with its own fluid, having allowed it to remain a short time in the basin.

August 4th. The case went on in every respect as those injected with wine and water; the inflammation fully sufficient, yet moderate.

I next give a curious case of supposed hermaphroditism.

Case IX.—*Tumors mistaken for Inguinal Hernia.*—January 31, 1828. Miss W——, aged 26, applied three months ago concerning a tumor in the left groin, which her mother told me was a rupture, and I believed it to be one, and advised a truss; found great difficulty in getting a truss to fit. Last month she applied concerning a similar though smaller tumor on the right side, which I examined two or three different times without being satisfied as to its nature.

This day I find the tumor on left labium, to which the truss was applied, to be about the breadth of three fingers in its long diameter, that of the right side the breadth of two fingers in its length; they are both very movable, and can be pushed down more than one half the length of the labium without any pain; but on attempting to force either into the ring she suffered much pain. The tumors are smooth, elastic, as if fluid were contained in a firm sac; in walking she feels no uneasiness at first, afterwards they become uneasy; in coughing no protrusion is felt against the fingers applied to the inguinal rings. General health good.

I suspect the tumors to be testes.

June, 1832. Tumors smaller than male testes; the vessels of the cord can be distinctly felt; squeezing them causes uneasiness; the vagina can scarcely admit the finger, not more than an inch in length. She admits that she never menstruated. In place of the male penis was a body resembling the glans penis, except, on turning it up, it is found divided in its entire length: the urethra is seen at a considerable distance from this.

Case X.—*Inguinal Hernia.*—January 29th. Assisted in operating on a young man, aged 20, for inguinal hernia of the right side. There was a great doubt as to the nature of the disease, for the tumor most strikingly resembled hydrocele of the tunica vaginalis, and the test of the candle showed it to be quite transparent. Still, I was satisfied that the intestine was down, and not to be returned by taxis. The sac was very thick; it proved to be congenital hernia. On passing the finger into the neck of the sac it was found to pass up very high before reaching the abdomen; high up in this was the stricture; being divided, the bowels were easily returned.

I would now direct attention to a few cases where there were doubts as to the nature of the disease, as to whether it was hernia or hydrocele, and even an operation undertaken for the relief of the symptoms.

Case XI.—*Hydrocele resembling Hernia.*—April 28, 1827. A middle-aged man presented himself with a hydrocele, large and oblong, being at one point more prominent to the eye and thin to the touch; it rose up so high along the cord as to resemble hernia. On pressing it while he stood, a part seemed to return with the sensation of an intestine. When laid horizontally, I pushed up the whole, and found the ring so wide as to admit two fingers. I now made him stand up while the fingers were in the ring; the tumor gradually returned below, yet the fingers did not feel anything passing down. On his coughing, the tumor descended with the same sort of burst as that of an inguinal hernia. I drew off three and three-quarters imperial pints of straw-colored fluid.

CASE XII.—*Hydrocele mistaken for Hernia.*—Monday, January 12, 1824. Called by Mr. R—— to a child 18 months old. He told me he had been often called to replace a congenital inguinal hernia; generally he succeeded, but as the testes had not descended, a truss could not be applied; now he could not succeed; the intestine was down since Saturday. I could get only a confused account from the nurse; neither the state of the abdomen, the countenance, nor the stomach, seemed to require immediate operation; besides, I had my doubts of hernia, for, on examining the tumor, I felt at the lower part a body which I suspected to be the testis, nearly double the size of the opposite. On passing the finger and thumb to the ring, I did feel the tumor as passing up through the opening into the abdomen. However, Mr. R——'s assertions prevailed. Calomel, half a grain every hour, and enemas.

At 11 o'clock at night I was called on to see the child, who was much worse; vomited everything, and had no stool, except some hardened fæces from enemas; the belly was not painful to the touch, nor swollen; face pale, and eyes sunken. Under these circumstances I agreed to the operation.

Mr. R—— cut cautiously, and spent time in separating the coverings of the sac; laid bare the parts near the ring, and now was certain no intestine was in the opening; he opened the lower part and let out water; slit up the sac on a director, and saw the testis lying about the middle of the sac; the director was then passed up the canal into the abdomen; no intestine was there. The wound was united by stitches and plaster.

Wednesday. Going on well. Had griping.

Had I been called alone to this case, I could not have suspected strangulated hernia from the feel of the parts. The test of a candle was not admissible, from fat and flatness; fluctuation could not be felt from tenseness of the tumor.

January 30th. Completely recovered from the operation.

CASE XIII.—*Operation for Hernia in case of Inflamed Testis.*—November 27, 1806. Saw a patient of Mr. ——, a man aged about 40; his countenance sunken; a dark circle around the eyelids; great tossing; pulse weak and small; skin cold; frequent vomiting, hiccup, pain in the right side of the abdomen, which was not tense, but was sore to the touch. There was a tumor in the right groin less than an egg; near the pubes the integuments were red; it resembled a venereal bubo; the right testis was small, and could not readily be discovered. He said he sometimes pushed up the testis into the abdomen with the intestines; there was great difficulty to ascertain if it were femoral or inguinal hernia, as we could not satisfactorily feel the ring; on trying the taxis a gurgling noise of a portion of gut was heard, but we could not get up the hernia. Tobacco enema caused a desire to stool, but the enema did not pass; the vomiting was without any straining; it was merely a slight effort to spirt out from the mouth; he ejected about half a pint at each effort.

Mr. —— operated; cut down on the sac; on pinching it up, he felt another body within; cut a small hole and divided the sac; intestine appeared healthy; felt at the ring like a thick cord; divided the pillar of the ring, and found a second structure, which he divided; the intestine kept by adhesion; this was divided; and it then returned. He died the next day, suffering great pain in the abdomen.

On post-mortem examination the wound was emphysematous; the intestines were filled with liquid fæces, inflated, inflamed, and adhering to each other and to the peritoneum; found a body blocking up the abdominal ring and adhering to it. Our surprise was great to find it to be the right testicle.

Lastly, I record two cases of hernia occurring in unusual positions.

CASE XIV.—*Hernia on Back.*—May 9, 1829. I saw a daughter of Mr. L——, three years old; of delicate form, but in good health. Her father states that this tumor was observed at the time of her birth. It was then smaller than at present. Now the tumor is as large as a moderate-sized watch, more conical; on the back, immediately above the left spine of the ilium, it admits of being easily returned, but quickly appears again. She has been away from her parents for the last three months, during which time they think her figure is slightly deformed, for there is a fulness along the right side of the spine, and on the left side, on a line with and close to the tumor, there seems to be a concavity. She has perfect use of her limbs.

CASE XV.—*Unusual Ventral Hernia.*—May, 1829. Mr. W—— has intestinal hernia, as large as a watch on the right side, half a finger's breadth from the spine of the ilium, and a finger's height above Poupart's ligament; this he retains in the abdomen by means of a leather bandage with a pad attached.

The subject of diseases of the rectum occupied much attention, and he gave his opinions on the subject in a paper in the Dublin Hospital Reports. I also publish another paper of his, with cases of disease of the rectum. The remaining cases recorded I have endeavored to collect and arrange in a condensed form.

I find, from the year 1823 to 1841, 69 cases noted; the youngest 4 years old, the eldest 70; of these, 32 were females, and 28 males; 15 had vascular tumors; 38 were affected with ulcers or fissures; 10 had both vascular tumors and fissures. In cases of tumors he applied caustic to one, and cut the remainder, pulling down the tumor and removing it with a scissors close to the forceps. Of these, 2 had hemorrhage; in 1 the bleeding occurred, as reported, from cutting too high. We should never cut above the sphincter. The bleeding was arrested by a sponge. In the second case of hemorrhage the report is: "I found no difficulty in drawing down the integuments, and securing the bleeding vessel."

These are the only unfavorable cases recorded, though he must have operated on a great number of cases, of which no mention is made, as they were unattended with any remarkable occurrence or symptom.

In 38 of these cases the disease was fissure or ulcer: of these, he operated by incision in 21, and he applied caustic, nitrate of silver, caustic potash, or sulphate of copper, to 16. Some of these required a second or third application of caustic, but these seemed to have all terminated favorably.

Of cases where there was the combination of vascular tumor and fissure, there are 12 recorded: of these he treated 8 by incision of the ulcer, and 4 by caustic; of these, 1 was attacked by erysipelas, and recovered; 1 died, and an abscess was found beneath the rectum.

I find, in fine, some cases of wounds of the brachial artery, and aneurisms consequent on bleeding, to the number of twelve.

They occurred at different ages. They came under notice from three days to six weeks from the time of the bleeding; in the majority the wound was open, and the patient reduced by repeated bleedings. In only two were the wounds closed; many of them appear to have had a sac with an opening in the artery, as well as externally.

In one remarkable case the patient was bled for an injury of his head; he seemed to progress favorably for some days, when an abscess formed under the cut, and opened, and then commenced to pour out blood.

One was a case of aneurismal varix, in which the patient compared the noise in the tumor to the hissing of a butterfly. Ten were operated on, and after more or less trouble a ligature passed above and below the wound.

In one a ligature high up did not control the bleeding, but, placed on the same vessel lower down, effectually stopped the flow from the upper portion.

In two there were more than two ligatures; in one, four different ligatures were applied, after various dissections, before the bleeding was stopped, each vessel being supposed to be the one injured.

In one case a ligature was applied above the wound. Secondary hemorrhage occurred in two days, and required a second operation to secure the lower end of the vessel.

In the aneurismal varix there was secondary hemorrhage on the eighth day, but it stopped without any further interference.

There was one case which had a fatal result; the patient died from the repeated bleedings, being exhausted by secondary hemorrhage.

The ligature came away in all of those recorded, from the 12th to the 23d day.

Two cases of aneurism were cured by pressure made on the tumor by means of a fine sponge. The first occurred in 1813.—*Dublin Quarterly Journal of Medical Science, May*, 1857.

ON THE CONCRETIONS OF THE PROSTATE. By *H. Thompson.*—The existence of "concretions" of microscopic size has been established in every one of the fifty specimens of the prostate exhibited. In many their size was that of a poppy-seed. They had been found also in the organ at fourteen years of age. Their physical and chemical characters (the latter by rigid analysis), were given at considerable length. Their existence was concluded to be a necessary result of the performance of natural functions on the part of the prostate. After numerous observations it appeared that the formation of a concretion frequently originated in the aggregation of a yellowish matter, often seen within the secreting nuclei lining the gland-ducts and pouches, often found free in yellowish granules, sometimes stuffing small ducts and follicles, and seen floating in the form of small prostatic fluid as well as in the contents of the vesiculæ seminales. In the larger masses of this yellow matter, entirely occupying the interior of crypts or follicles, the small granules may be seen cohering, or as if fusing together, and presenting an appearance identical with that which is often seen existing in the centre of fully formed concretions. It was concluded that the coalescence of these yellow granules, or of the nuclei charged with them, their partial fusion into a mass more or less homogeneous, the stratification in part of this mass itself, or more probably the deposit upon its surface of fresh layers of fluid matter similar to that which originally constituted the interior, and finally, some addition of opaque earthy matter to it, either by infiltration or accretion (through irritation of the secreting membrane around, from the pressure of the newly-formed body, as observed in numerous other instances referred to), were the steps by which the production of a "prostatic concretion" was very frequently accomplished, and its connection with "prostatic calculus" illustrated. The views of other observers were quoted and discussed at considerable length. Numerous drawings of these bodies in various stages of formation, as well as the original objects themselves under microscopes, illustrated the communication.—*Medical Times and Gazette*, June 20, 1857.

